# Shear Behavior of Reinforced Concrete Beam Retrofitted with Modularized Steel Plates

**DOI:** 10.3390/ma16093419

**Published:** 2023-04-27

**Authors:** Min Sook Kim, Young Hak Lee

**Affiliations:** Department of Architectural Engineering, Kyung Hee University, Deogyeong-daero 1732, Yongin 17104, Republic of Korea; kimminsook@khu.ac.kr

**Keywords:** retrofitting, modularized steel plates, shear behavior, shear span-to-depth ratio, shear strength equation

## Abstract

This paper presents the results of a shear test on a reinforced concrete beam retrofitted with modularized steel plates. A total of five retrofitted concrete beams with various span-depth ratios as a variable were fabricated and tested. A companion beam without retrofitting was used as the control specimen. The results of this experiment confirmed that the method proposed in this study improved the shear performance by approximately 1.8 times compared with the non-retrofitted reinforced concrete beam. The test results indicate that the shear retrofitting method using modularized steel plates can be effective in retrofitting the concrete beams, resulting in improvement in the strength, stiffness and deformations.

## 1. Introduction

**Figure 1:**
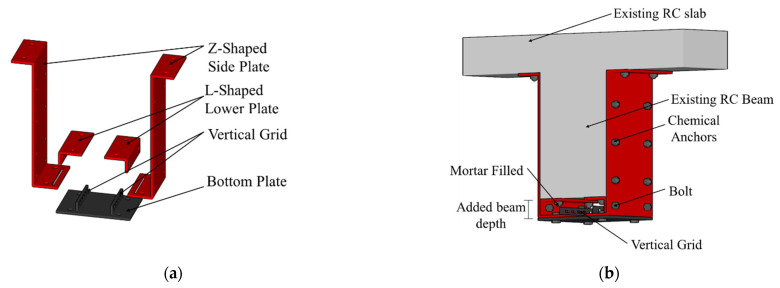
Schematics of the proposed retrofitting system [16]. (**a**) The components of the retrofitting system; (**b**) RC beam retrofitted with modularized steel plates.

In reinforced concrete (RC) structures, brittle failure can be caused by a lack of strength and ductility if the RC structure is designed without seismic details or if degradation occurs due to aging of the RC structure. Therefore, to maintain the function of RC buildings, structural safety should be checked periodically, and retrofitting methods that can enhance the strength and ductility of existing structures should be applied. The most common retrofitting method of RC structures is steel jacketing to confine the concrete columns or beams [1,2,3]. However, there are difficulties in retrofitting beams using steel jacketing such as lifting the steel plates. Therefore, studies on steel retrofit methods for beams have focused on ways to reduce the amount of steel and improve the strength and ductility by using high-performance hybrid materials such as fiber-reinforced polymers (FRP) [4,5,6,7,8], or facilitating constructability [9,10,11]. In methods involving bonding a steel plate or FRP, deboning failure may occur due to degradation or debonding of a bonding material, and particularly, when FRP is used, the fracture strain of the material itself is small and ductility is low, and brittle failure may thus occur [12,13,14,15]. Therefore, the authors proposed a modularized retrofitting method for RC beams in the previous study [16], as shown in Figure 1.

The steel plates bonded to the beam are modularized so that they can be bonded to the side and bottom surface of the beam separately, and each steel plate is bonded with chemical bolts for facilitating lifting and construction. The modularized steel plate retrofitting method consists of two Z-shaped side plates, two L-shaped lower plates, and a bottom plate; the side plates and lower plate are bonded to the side and bottom of the beam, respectively, and the bottom plate is welded with a vertical grid that enhances flexural performance.

The construction process is as follows:

(1) The L-shaped lower plates are fixed to the bottom of the concrete specimen, using a chemical anchor.
(2) The Z-shaped side plates are bolted to the L-shaped lower plates and then bonded to the side of the beam with a chemical anchor.
(3) The bottom plate is inserted into the slot of the Z-shaped side plates to tighten the bottom plate and bond with bolts.
(4) After integrating the side plates with chemical anchors, mortar is injected between L-shaped lower plates and the bottom plate, while the specimen is upside down to prevent filling defects in the mortar.

All components are modularized and connected by bolts, so this method can be installed regardless of the size of the beams, and additional lifting equipment is unnecessary. In addition, it can maintain constant quality regardless of the efficiency of the workers. Since a bottom plate acting as tensile reinforcement and stirrup is installed, a subsequent process of installing excessive reinforcement on the tensile zone is not needed. The steel plate is attached to the existing beam using chemical anchors and mortar, so they are effectively integrated.

Attaching steel plates to beams has been used for decades. It has been mainly applied in two ways: partially on the tension surface to enhance flexural performance, or partially on the sides of the beam to enhance shear performance. The objective of this paper is to evaluate the retrofitting technique of attaching steel plates to the vertical sides and bottom of the beam to improve their shear performance. The steel plate that attaches to the beam is modularized in each part and installed with bolts and anchors. This reduces the weight of the steel plates, making it easier to install. After evaluating the flexural performance of the modularized steel plates retrofit method described in previous research [16], a shear experiment was conducted by fabricating one non-retrofitted specimen and five retrofitted specimens to evaluate the shear performance. The applicability of shear strength equations specified in the ACI 318-19 [17] and proposed from the many studies [9,18,19] to retrofitted RC beams using the proposed method was evaluated.

## 2. Experimental Program

### 2.1. Specimen Details

**Figure 2:**
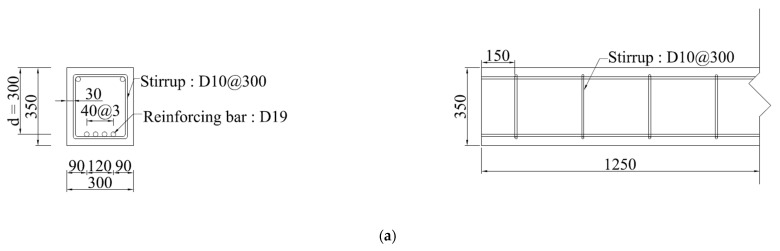

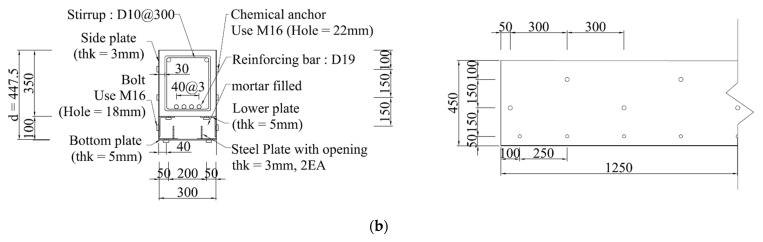
Specimen details (mm). (**a**) RC beam specimen (RC-2); (**b**) specimen retrofitted with modularized steel plates.

In this paper, to analyze the shear behavior of the proposed retrofitting method, one RC beam specimen and five retrofitted RC beam specimens with modularized steel plates were fabricated. The shear behavior was evaluated according to the shear span-to-depth ratio of RC beams retrofitted with steel plates, using the shear span-to-depth ratio as a variable. Details of the specimens are summarized in Table 1. Previous research [16] proposed a Z-shaped steel plate capable of wrapping both the bottom and side of the beam to fix the steel plate bonded to the beam to the slab. In in situ applications, the beams would be supporting the slab, but in the experiment in this study, slabs were not fabricated in the same way as in the prior study [16] because slabs are used only to fix the steel plates. Drawings of the specimens are shown in Figure 2. The RC beams of all specimens were designed to have a 300 mm width, 300 mm effective depth, and 2500 mm length. The depth of the new retrofitted beam was 100 mm. Since the steel plate covers the entire beam, the steel plate at the bottom of the beam also contributes tensile force without spalling of the concrete cover. Therefore, the effective depth of the retrofitted beam was determined by subtracting 1/2 of the bottom plate thickness from the total height of the beam as shown Figure 2b.

The bonding performance between modularized steel plates and integrated behavior between modularized steel plates and existing RC beam were verified through previous research [16]; this study used a modularized steel plate of one span length to simplify the fabrication and construction process, unlike the previous research. To prevent flexural failure before shear failure, four tensile rebars with diameters of 19 mm were arranged. The spacing between the chemical anchors or bolts is 300 mm. The thicknesses of the vertical grid with an opening and the side steel plate were 3 mm, and the lower plate and bottom plate were 5 mm each. The diameters of the high-tensile bolt used for the bonding between steel plates and the chemical anchor used for bonding the steel plates to the existing beam were 16 mm each.

**Figure 3:**
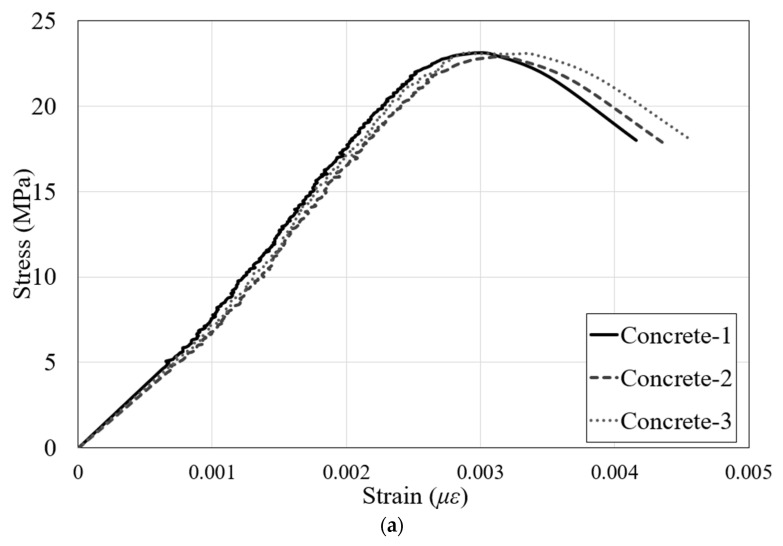

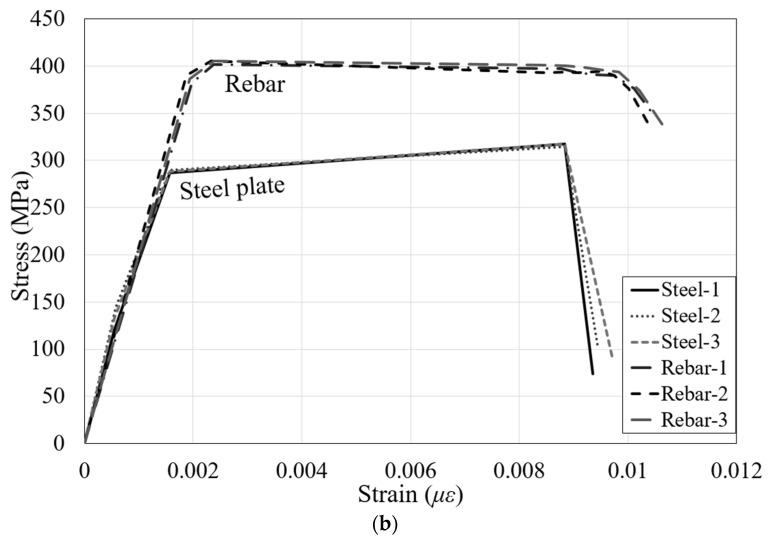
Material property tests. (**a**) Compressive strength test of concrete; (**b**) tensile test of rebar and steel plate.

The material properties of the specimens were determined by averaging the experimental results of three specimens according to the Korean standards as shown in Figure 3. The 28-day compressive strength of concrete was 23.2 MPa, and the yield strengths of deformed reinforcements and steel plates were 402.8 MPa and 275.5 MPa, respectively. The material properties are listed in Table 2. To verify the validity of the experiment conducted in this study, two specimens with the same specifications and shear span-to-depth ratio were fabricated, and the specimens were named RSB-2A and RSB-2B.

**Figure 4:**
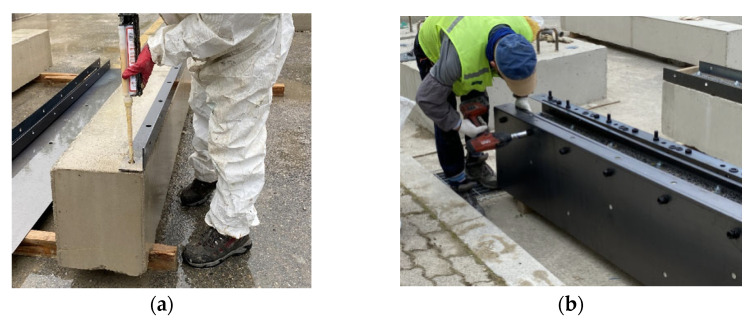

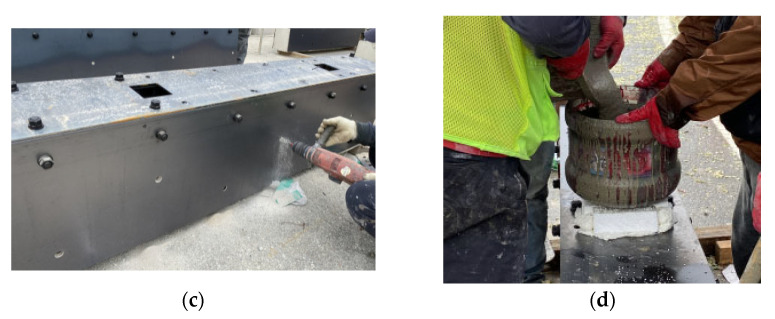
Retrofitting process. (**a**) Installation of L-shaped lower plate; (**b**) connection of Z-shaped side plate and bottom plate using bolts; (**c**) installation of Z-shaped side plate using chemical anchors; (**d**) grouting of mortar.

The construction process of the test specimens is shown in Figure 4. After scraping the concrete surface, L-shaped lower plates were fixed to the bottom of the RC beam with chemical anchors. The L-shaped lower plate and Z-shaped side plate were bolted, and the bottom plate was inserted to fasten it and the Z-shaped side plate with bolts. The Z-shaped side plate was connected to the side of the concrete with chemical anchors, and mortar was injected into the newly established region using the steel plates.

### 2.2. Test Set Up

**Figure 5:**
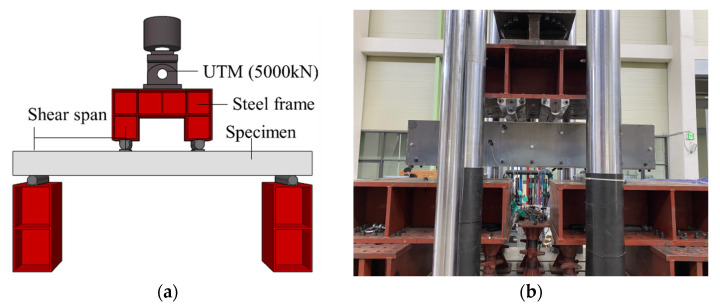
Test setup. (**a**) Schematic test setup; (**b**) photograph of test setup.

**Figure 6:**
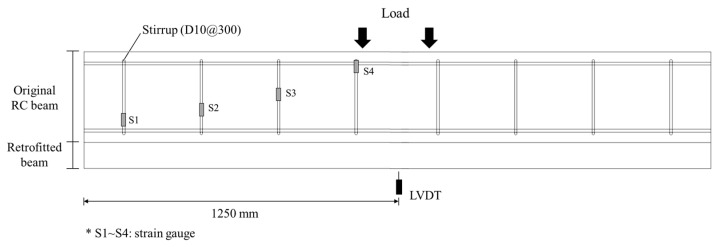
Location of LVDT and strain gauges (mm).

As shown in Figure 5, the shear experiment was conducted by applying a load to the simply supported specimen at rate of 2 mm/min using a hydraulic universal testing machine (UTM) with a capacity of 5000 kN. The spacing between the loading points of all specimens was equal (400 mm), and the shear span-to-depth ratio was adjusted by changing the shear span. The experiment was conducted until the load decreased to between 70% and 80% of the maximum load. To measure the deformation of the specimens, linear variable displacement transducers (LVDTs) were mounted onto the bottom of the specimens, and to investigate the shear behavior, strain gauges were bonded to the stirrups. Strain gauges were bonded to the surfaces of the steel plates on the retrofitted specimens. The installation of the LVDTs and strain gauges is shown in Figure 6.

## 3. Test Results and Discussion

### 3.1. Crack Propagation and Failure Mode

**Figure 7:**
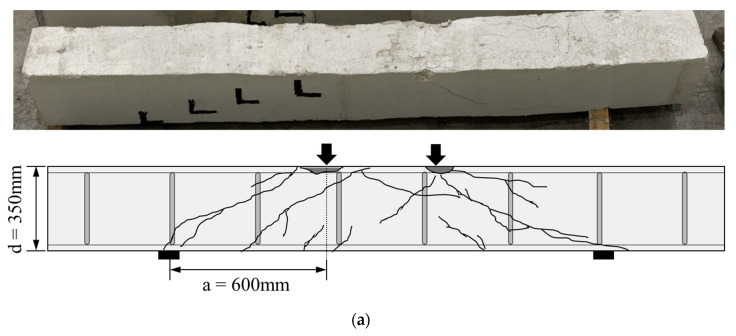

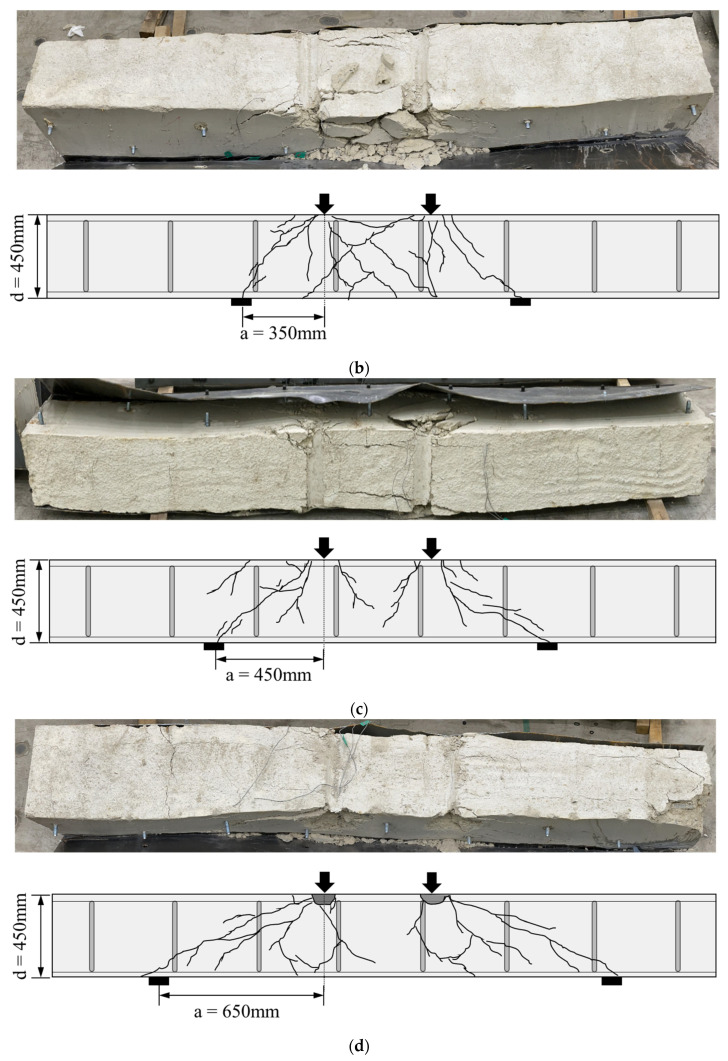

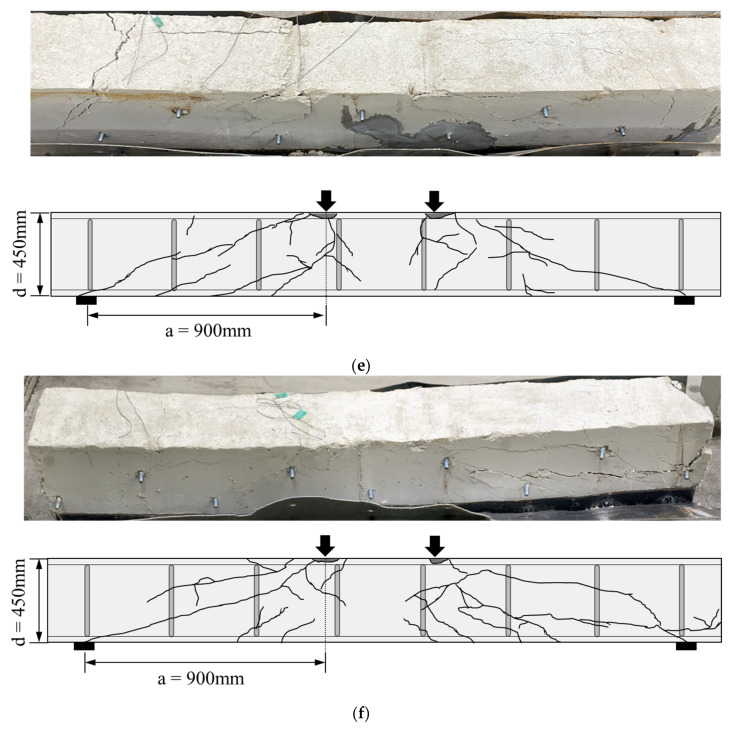
Crack patterns. (**a**) RC-2; (**b**) RSB-0.8; (**c**) RSB-1; (**d**) RSB-1.5; (**e**) RSB-2A; (**f**) RSB-2B.

**Figure 8:**
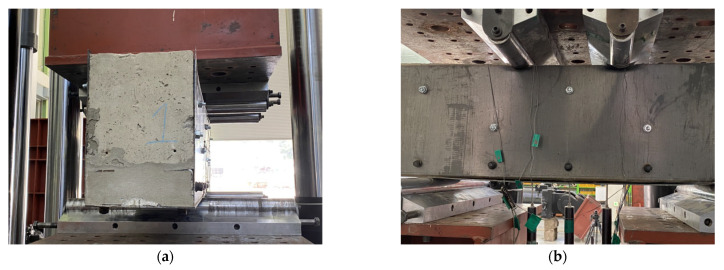
Modularized steel plates after a shear test. (**a**) Side view; (**b**) front view.

The propagation of cracks in all specimens is shown in Figure 7. In the case of specimens retrofitted with modularized steel plates, steel plates were removed after the experiment was terminated to observe crack propagation in the concrete. In RC-2, the first crack was observed in the form of the flexural crack in the central span at the beginning of the loading. However, as the load increased, the shear dominant behavior formed shear cracks as shown in Figure 7a. Therefore, Figure 7a shows that shear cracks were predominantly observed. As the load increased, shear cracks developed from the supports to the loading points, and the concrete was crushed in the compressive zone. In the case of specimens retrofitted with modularized steel plates, the crack propagation in the concrete according to the loading stage could not be observed due to the steel plate, but it was confirmed that all specimens had failed, showing that shear cracks developed from the support and loading points, as shown in Figure 7b–f. The RSB-0.8 and RSB-1 had very small shear span-to-depth ratios (less than 1), and a number of cracks extending from the support to the loading point were observed. It was confirmed that shear cracks of the RSB-1.5, RSB-2A, and RSB-2B developed in the concrete compressive zone, and shear-compression failure occurred due to the crushing of the concrete compressive zone. This is because the shear strength is greater than that of the tensional diagonal cracks. Figure 8 shows the joints between the steel plates and the concrete surface. When the experiment was terminated, there was no local buckling at the side steel plate and bottom steel plate for all specimens, and there was no debonding between the existing RC beam and the side steel plate. Therefore, it is considered that the retrofitting method was integrated with the existing beam.

### 3.2. Load-Deflection Relationship

**Figure 9:**
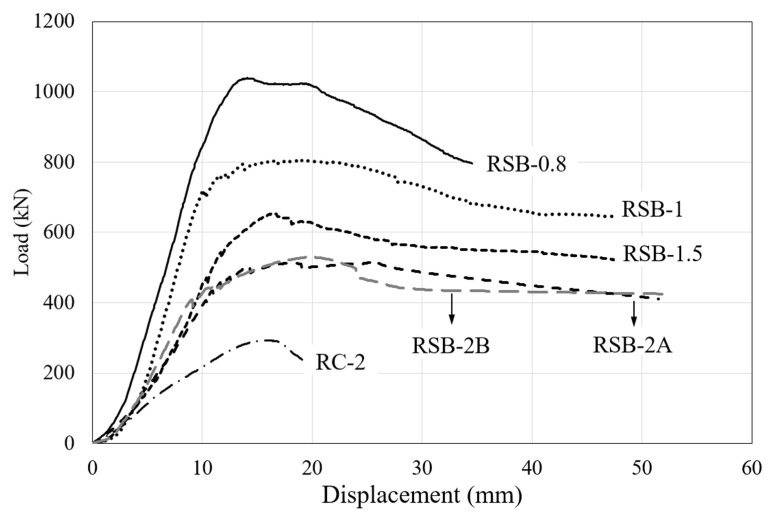
Load–displacement curve.

**Figure 10:**
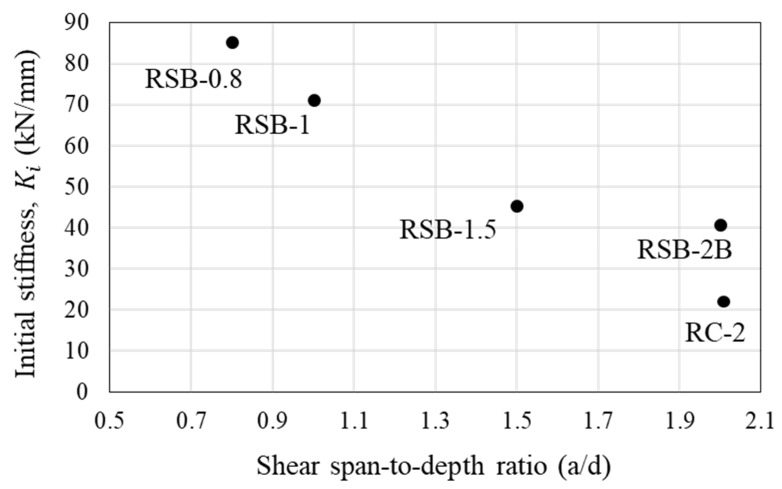
Effect of shear span-to-depth ratio on initial stiffness.

The test results are summarized in Table 3, and the load–displacement curves of each specimen are shown in Figure 9. To verify the validity of the experiment conducted in this study, RSB-2A and RSB-2B were fabricated with the same details, and their behavior was investigated in the same manner. The experimental results confirmed the validity of the experiment because both the crack patterns and the load–displacement relationship between the two specimens were similar. Therefore, this study analyzed the experimental results of RSB-2B, which had the greater maximum load value of the two specimens. It was confirmed that the maximum load of RSB-2B was 529.25 kN, which is 1.8 times higher than the maximum load of non-retrofitted specimen RC-2. In addition, the initial stiffness of RSB-2B was 1.9 times that of RC-2, and it was confirmed that the proposed method improved the stiffness of the RC beam. Therefore, the proposed method of retrofitting steel plates is considered to effectively improve the shear performance of the RC beams. In general, the shear span-to-depth ratio can significantly affect the shear capacity of RC beams. As shown in Figure 10, the experimental results of retrofitted specimens whose shear span-to-depth ratio is a variable verified that the maximum load and initial stiffness decreased as the shear span-to-depth ratio increased. This is because the bending deformation of the member increases as the shear span-to-depth ratio increases, so the arch action decreases and cracks in the shear span increase. Through this, it was confirmed that the shear span should be considered as a variable when the shear capacity of RC beams retrofitted by the proposed method is evaluated. However, as shown in Figure 10, the initial stiffness decreased from 85.23 kN/mm to 45.34 kN/mm as the shear span-to-depth ratio increased from 0.8 to 1.5, and the initial stiffness decreased from 71.29 kN/mm to 42.10 kN/mm as the shear span-to-depth ratio increased from 1.0 to 2.0. It can be confirmed that the initial stiffness of a beam retrofitted with the steel plates is significant when the span-to-depth ratio is less than 1.5, indicating that the span-to-depth ratio should be considered in the design of retrofitting for concrete beams subjected to high shear force such as deep beams.

### 3.3. Load–Strain Relationship

**Figure 11:**
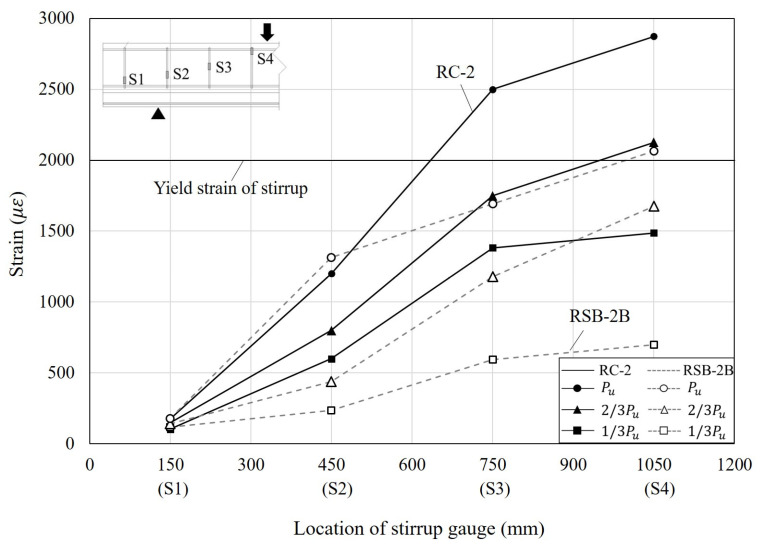
Comparison of strain as a function of stirrup gauge location.

**Figure 12:**
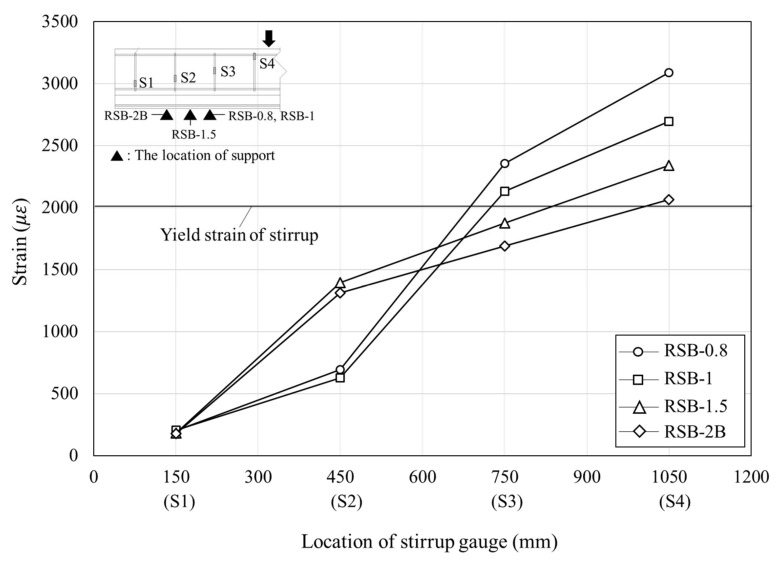
Relationship between shear span-to-depth ratio and stirrup strain.

To analyze the shear behavior of the RC beam, the strain of the stirrup, which is resistant to shear force, is the most important factor. Therefore, the strain of the stirrup was observed according to load increment by attaching gauges to stirrups to analyze the shear behavior for the retrofit method proposed in this study. Figure 11 indicates the strain distributions of the stirrups in RC-2 and RSB-2B, which have the same shear span-to-depth ratio at different load levels. In Figure 11, the x-axis represents the location of the stirrups (the distance to the section from the left support), whereas the y-axis represents the measured strain values. In the case of both specimens, the strain measured by gauge S1 was quite small since it is located between the support and the loading point, and the strain increased from gauge S2 to gauge S4 as it approached the loading point from the support. In the case of RC-2, gauge S4 yielded when the load was two-thirds of the maximum load, and gauge S3 yielded as the load approached the maximum load. However, in the case of RSB-2B, gauge S4 yielded as the load approached the maximum load. In addition, at the same load level, RC-2 showed a greater stirrup strain than RSB-2B. It is considered that the vertical grid welded to the lower plate and the Z-shaped side plate of RSB-2B distributed the shear force. In the case of RC-2 and RSB-2B, their S4 gauges showed strains of 0.00287 and 0.00206, respectively, when the load reached the maximum load, and they increased by 35% and 23%, respectively, relative to the strains when the load was two-thirds of the maximum load. Through this analysis, it is considered that the modularized steel plates retrofit method in this study can prevent brittle failure of the members and effectively resist shear force. Figure 12 shows the strain at the maximum load. It was confirmed that all specimens showed a larger strain on the stirrups located at the loading point, and the stirrups yielded. This is because shear cracks occurred from the support to the loading point, and as the load increased, they propagated to the concrete compressive zone surface and the members reached failure. The strain increased significantly as the shear span-to-depth ratio decreased. This is because the maximum load increased proportionately as the shear span-to-depth ratio decreased. However, since gauge S2 is not located between the support and the loading point in the case of RSB-0.8 and RSB-1, the strain at gauge S2 of RSB-1.5 and RSB-2B was about three times greater than that of RSB-0.8 and RSB-1.

### 3.4. Prediction of Shear Strength

This study evaluated the shear strength of the RC beam retrofitted with modularized steel plates. It can be calculated by summation of shear strengths of concrete and shear reinforcement of existing RC beam and Z-shaped side plate as shown in Equation (1). Since no de-bonding steel plate was observed, it was assumed that the steel plate and concrete beam were perfectly bonded. The equation of shear strength of concrete proposed by Zsutty [17] was used to consider the effect of the shear span-to-depth ratio as shown in Equation (2). In order to calculate the shear strength of the stirrup, Equation (3) provided by ACI 318-19 [18] was used.

(1) $$V_{n}=V_{c}+V_{s}+V_{sp}$$

(2) $$V_{c,Zsutty}=2.17{\left({f_{c}}^{\prime }\rho_{w}\frac{d}{a}\right)}^{1/3}b_{w}d$$

(3) $$V_{s}=\frac{A_{v}f_{yt}d}{s}$$

where $V_{n}$ is the shear strength of the reinforced concrete beam, $V_{c}$ is the shear strength of concrete, $V_{s}$ is the shear strength of shear reinforcement, $V_{sp}$ is the shear strength of the steel pate, $A_{v}({mm}^{2})$ is the area of shear reinforcement within spacing, $b_{w}$ is the web width, d is the effective beam depth, $\rho_{w}$ is the ratio of $A_{s}$ to $b_{w}d$, $f_{yt}(MPa)$ is the specified yield strength of transverse reinforcement, $f_{y}$ is the specified yield strength of steel, and s is the center-to-center spacing of transverse reinforcement.

To consider the shear strength of the Z-shaped side plates proposed in this study, the existing shear strength equations of the steel plate were used as shown in Equation (4) in AISC 360-16 [19] and Equation (5) proposed by Altin et al. [9]. The shear strength equation of Altin et al. [9] considered the bonding of a steel plate to concrete using epoxy. The average shear stress of the epoxy for specimens strengthened with steel plate for this study is 0.8 MPa as they suggested.

(4) $$V_{sp,AISC}=0.6f_{y}A_{w}$$

(5) $$V_{sp,Altin}=2\left[\tau_{ave}\left(\frac{dh_{W}}{2}\right)\right]$$

where $A_{w}$ is the effective area of the web, $\tau_{ave}$ is the average shear stress of the epoxy, and $h_{W}$ is the height of the steel plates.

**Figure 13:**
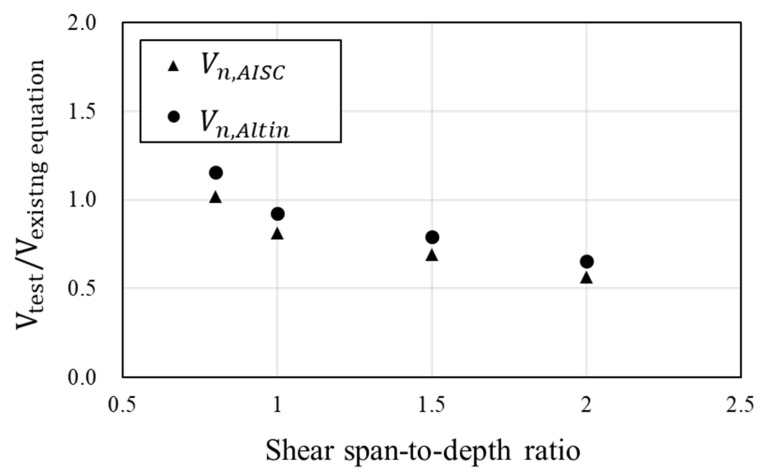
Comparison of test results with values calculated using existing shear strength equations.

Comparison of these approaches with the test results is presented in Table 4 and Figure 13. The modularized steel plate is bonded to the RC beam over the entire span, the degree to which it contributes to the shear strength varies depending on the shear span-to-depth ratio. Overall, AISC and Altin’s equation tends to overestimate the shear strength. As the shear span-to-depth ratio increases, the shear strength of the experiment results decreases significantly, but the predictions do not decrease as much. This is because the predictions assume that the steel plate resists shear equally across the span of the beam. They only calculate the area of steel plate. Therefore, only the steel plates in the region where the compression zone is formed within the shear span should be considered as contributing to the shear resistance.

## 4. Conclusions

To evaluate the shear performance of RC beams retrofitted with modularized steel plates, five retrofitted concrete beams were tested. The crack patterns, load–displacement relationships, stiffness, and strains were analyzed. The applicability of the existing shear strength equations to the retrofitting method was also evaluated. The following conclusions can be drawn from this study:

(1) Typical shear dominant cracks and failure modes were observed in all specimens. The failure of the retrofitted beam in shear is characterized by a gradual and ductile behavior in contrast to the typical brittle failure of RC beams in shear. This means that the modularized steel plates contribute to the ductile behavior. At the end of the experiment, local buckling did not occur at the side plates and bottom plate, and the joint between the existing RC beam and side steel plate was not detached. Therefore, the spacing and bonding method of anchor bolts ensures the attachment of the concrete beam and the steel plates.
(2) It was confirmed that the maximum load of RSB-2B was 529.25 kN, 1.8 times the maximum load of RC-2. In addition, the initial stiffness of RSB-2B was 1.9 times that of RC-2, and it was also confirmed that the retrofit method with modularized steel plates improves the stiffness of the RC beams. Therefore, the retrofitting method in this study is considered to effectively improve the shear performance of RC beams. As the shear span-to-depth ratio decreased, the maximum load increased, and then after reaching the maximum load, it gradually failed. The steel plates improve the shear strength and contribute to preventing brittle failure.
(3) This study evaluated whether the shear strength of RC beams retrofitted by modularized steel plates can be predicted by the existing shear strength equations including the AISC 360-16 and ACI 318-19 provisions and the proposed equation by the other researchers. As the comparison of the experimental results with the existing shear strength equations shows, they overestimated the experimental shear strength with a large shear span-to-depth ratio. This is due to the assumption that the steel plates installed throughout the beams contributed equally to the shear resistance. It is recommended that the existing shear strength equations be modified to account for the actual contribution of the steel plates to the shear resistance within the shear span.

## Figures and Tables

**Table 1:** Test specimens.

No.	Name	Retrofit Method	Added Beam Depth (mm)	Shear Span-to-Depth Ratio (a/d)
1	RC-2	None	None	2
2	RSB-0.8	Modularized steel plates	100	0.8
3	RSB-1		100	1
4	RSB-1.5		100	1.5
5	RSB-2A		100	2
6	RSB-2B		100	2

**Table 2:** Material properties.

Material	Compressive Strength (MPa)	Yield Strength (MPa)	Tensile Strength (MPa)	Modulus of Elasticity (MPa)
Concrete	23.2	-	-	25,563
Rebar, stirrup	-	402.8	-	200,000
Steel plate	-	275.5	316.4	205,000

**Table 3:** Test result.

No.	Name	$P_{u}$ (kN)	$P_{0.8u}$ (kN)	$\delta_{u}$ (mm)	$\delta_{0.8u}$ (mm)	$K_{i}$ (kN/mm)
1	RC-2	293.40	234.90	15.41	19.35	22.16
2	RSB-0.8	1038.95	831.45	14.08	31.93	85.23
3	RSB-1	805.40	644.30	18.99	47.57	71.29
4	RSB-1.5	654.10	523.20	16.83	47.19	45.34
5	RSB-2A	515.11	414.90	18.13	51.51	40.67
6	RSB-2B	529.25	423.40	19.56	52.30	42.10

$P_{u}$: maximum load of each specimen, $P_{0.8u}$: yield load of each specimen (0.8 $P_{u}$), $\delta_{0.8u}$: displacement when 0.8 $P_{u}$, $\delta_{u}$: displacement when maximum load is applied, $K_{i}$: initial stiffness.

**Table 4:** Comparison of shear strength.

No.	Specimens	Test	Existing Shear Strength Equation	
		$V_{test}$ (kN)	$V_{n,AISC}$ (kN)	$V_{n,Altin}$ (kN)
1	RSB-0.8	519.48	510.1	449.4
2	RSB-1	402.70	495.8	435.0
3	RSB-1.5	327.05	472.3	411.5
4	RSB-2A	259.33	457.4	396.6

where $V_{n,AISC}$ is shear strength of RC beam retrofitted with modularized steel plate using shear strength equation of steel plate in AISC 360-16 [19], and $V_{n,Altin}$ is using shear strength equation of steep plate proposed by Altin et al. [9].

## Data Availability

Not applicable.
